# Supplementary material for: Long-Term Oral Tamoxifen Administration Decreases Brain-Derived Neurotrophic Factor in the Hippocampus of Female Long-Evans Rats
Source: Cancers (Basel). 2024 Mar 31;16(7):1373. doi: 10.3390/cancers16071373 (PMC11010888; doi:10.3390/cancers16071373)
Supplement: Supplementary file 1 [file cancers-16-01373-s001.zip › cancers-2814333-supplementary.pdf]

Supplementary Figure S1: Body Condition Scoring

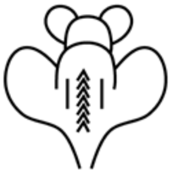

BC 1
Rat is emaciated
- Segmentation of vertebral column prominent if not visible
- Little or no flesh cover over dorsal pelvis, pins prominent if not visible
- Segmentation of caudal vertebrae prominent

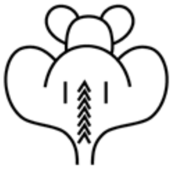

BC 2
Rat is under-conditioned
- Segmentation of vertebral column prominent
- Thin flesh cover over dorsal pelvis, little subcutaneous fat, pins easily palpable
- Thin flesh cover over caudal vertebrae, segmentation palpable with slight pressure

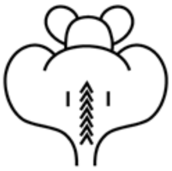

BC 3
Rat is well-conditioned
- Segmentation of vertebral column easily palpable
- Moderate subcutaneous fat store over pelvis, pins easily palpable with slight pressure
- Moderate fat store around tail base, caudal vertebrae may be palpable but not segmented

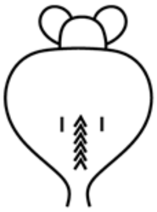

BC 4
Rat is over-conditioned
- Segmentation of vertebral column palpable with slight pressure
- Thick subcutaneous fat store over dorsal pelvis, pins of pelvis palpable with firm pressure

Supplementary Figure S1: Body Condition Scoring

- Thick fat store over tail base, caudal vertebrae not palpable

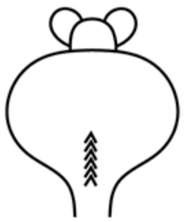

BC 5
Rat is obese

- Segmentation of vertebral column palpable with firm pressure, may be a continuous column
- Thick subcutaneous fat store over dorsal pelvis, pins of pelvis not palpable with firm pressure
- Thick fat store over tail base, caudal vertebrae not palpable

Adapted from: Hickman D, Swan M. 2010. Use of a Body Condition Score Technique to Assess Health Status in a Rat Model of Polycystic Kidney Disease, JAALAS 49(2) 155-159
